# Effectiveness of Pulmonary Rehabilitation for Chronic Obstructive Pulmonary Disease Therapy: Focusing on Traditional Medical Practices

**DOI:** 10.3390/jcm12144815

**Published:** 2023-07-21

**Authors:** Katrina O. Tonga, Brian G. Oliver

**Affiliations:** 1 Respiratory Cellular and Molecular Biology, Woolcock Institute of Medical Research, Macquarie University, Glebe, NSW 2037, Australia; katrina.tonga@woolcock.org.au; 2 Saint Vincent’s Hospital Sydney, Darlinghurst, NSW 2010, Australia; 3 School of Life Sciences, University of Technology Sydney, Sydney, NSW 2007, Australia

**Keywords:** COPD, pulmonary rehabilitation, exercise training, oxygen support, acupuncture

## Abstract

Chronic obstructive pulmonary disease (COPD) is a complex and serious disease that is characterized by dyspnea, fatigue, decreased exercise tolerance, peripheral muscle dysfunction, and mood disorders. These manifestations are successfully treated with pulmonary rehabilitation, a comprehensive intervention and holistic approach designed to improve the physical and psychological condition of people with COPD. Exercise is a big component of pulmonary rehabilitation programs, but the efficacy of non-traditional forms of exercise as used in alternative medicine is poorly understood. Here, we aim to address this gap in knowledge and summarize the clinical evidence for the use of traditional exercise regimens in the pulmonary rehabilitation of COPD patients.

## 1. Introduction

Chronic obstructive pulmonary disease (COPD) is a progressive lung disease that is a leading cause of chronic morbidity and mortality worldwide and creates a huge economic burden on patients and society. Cigarette smoking is considered as the major induction factor for COPD. The pathological mechanisms of COPD are not fully understood, but inflammatory mechanisms, oxidative stress, and protease–antiprotease imbalance are found as main factors involved in the development of COPD [1,2]. Drug intervention using antibiotics, inhaled corticosteroids, bronchodilators, and other medicines are widely used in clinical for COPD patients [3]. However, the side effects such as osteoporosis, heart rate disturbance, and increased probability of infection are frequently found with drug use [4,5]. Moreover, physical inactivity is common in COPD patients, resulting in an impaired health status and increasing rates of hospitalization and mortality [6]. On the basis of these current reasons, pulmonary rehabilitation has the greatest positive effect of any current therapy on exercise capacity in COPD, which appears to be particularly important.

According to the American Thoracic Society (ATS) and the European Respiratory Society (ERS), pulmonary rehabilitation is a comprehensive intervention based on a thorough patient assessment followed by patient-tailored therapies that typically consist of, but are not limited to, exercise training, education, and behavior change. The aim of this comprehensive intervention is to improve the physical and psychological condition of people with chronic respiratory disease and to promote the long-term adherence to health-enhancing behaviors [7]. Patients with chronic obstructive pulmonary disease (COPD) have lower muscle function and are less active [8,9], emphasizing the importance of pulmonary rehabilitation (PR) for COPD management. Indeed, pulmonary rehabilitation has been shown to improve a COPD patient’s physical capacity, dyspnea, fatigue, endurance capacity, and health-related quality of life [10]. Here, we summarized the main effects of pulmonary rehabilitation management in patients with COPD, focusing on treatment regimens originating in traditional medical practices.

## 2. Exercise Training Modalities for Pulmonary Rehabilitation

Exercise training is the cornerstone of pulmonary rehabilitation, and several models of exercise training have been performed on COPD patients to improve their whole-body exercise tolerance. In addition to traditional training modalities, Tai Chi, Yoga, Daoyin, and walking training have all been recommended as important components of many pulmonary rehabilitation programs [11].

Tai Chi is a Chinese recreational exercise that is based on yin and yang principles and includes a series of slow and rhythmic circular motions [12]. Numerous studies have found that Tai Chi improved the lung function, exercise capacity, health status, quality of life, and mental status in COPD patients and is recommended as an exercise regimen for people with COPD [13,14]. Tam et al. demonstrated that Tai Chi increased respiratory function, activity, as well as health status and alleviated the deterioration of COPD patients [15,16]. A study of 42 COPD patients who received supervised Tai Chi training twice a week for a total of 12 weeks found an increase in quadriceps muscle strength, balance, the incremental and endurance shuttle walk test, health status, and mood status [17]. Both Pan and Hu’s studies went on to provide the preliminary evidence in randomized clinical trials that Tai Chi enhances lung function and exercise capacity [18,19]. Moreover, in a meta-analysis, Tai Chi was found to enhance lung function, exercise capacity, health status, quality of life, and mental status of COPD patients [20].

Daoyin is a kind of mind–body exercise including Tai Chi, Qigong, and Baduanjin. It incorporates a series of designed movements and mind regulation with controlled breathing exercises. Li’s group suggested that pulmonary Daoyin is a suitable exercise option for COPD patients as it provides respiratory muscle training with special breathing. Specifically, through a randomized controlled study that included 464 COPD patients in 11 research centers in China, they conducted and demonstrated the efficacy of pulmonary Daoyin (PD) in patients with COPD. The results showed that the PD program was able to improve the activity tolerance level and satisfaction of COPD patients and enrich the traditional Chinese medicine rehabilitation technology of COPD [21,22]. This work was cited as "rehabilitation technology with Chinese characteristics" by *GOLD 2019*, providing the first international guidance and reference of PD application for COPD patients. 

Yoga originated from ancient India. It is a holistic approach including body posture, breath control, and concentration [23,24], in which it is said that the exercise may coordinate the individual self with the transcendental self [25]. A number of studies have shown the beneficial role of Yoga in COPD patients. In a short-term study, Schultz and his colleagues suggested that daily-based Yoga improved a COPD patient’s lung function and quality of life [26]. Additionally, Yoga-derived techniques for slower and deeper breaths have been reported to improve resting gas exchange which can induce favorable respiratory changes in COPD participants, with the more severe patients acquiring more benefits from this exercise intervention [27]. In coal miners with COPD, Yoga interference could reduce declines in dyspnea and fatigue and increase functional performance [28]. In COPD patients, dyspnea is considered as a major cause of depression, anxiety, and even disability [29]. A Yoga treatment regimen is reported to increase physiological measures of lung function in patients with COPD. For example, the transfer factor of lung for carbon monoxide (TLCO) was increased with yoga [30]. Consistently, Yoga training has declined dyspnea-associated negative psychologic well-being such as distress and anxiety [31].

Traditional training modalities including aerobic, resistance, and upper limb training have been showed to be beneficial among individuals with COPD. Endurance training of the lower limb muscles is the most commonly used aerobic training modality and is feasible in the PR program for patients following hospital discharge after an acute exacerbation of COPD [32]. Walking- and cycling-based exercise are preferred modes for endurance exercise training in the pulmonary rehabilitation for COPD patients. It is well studied that 3 months of the Nordic walking exercise program greatly improved walking intensity and the six-minute walking distance of COPD patients [33]. Another report illustrated that ground walking training significantly increased the endurance shuttle walk time [34]. Moreover, water-based walking seems to effectively improve the quality of life of COPD patients and those with physical comorbidities, and it is favorable for patients with obesity and/or arthrosis of the hip/knee/lower back [35]. While cycling training focuses on the quadriceps muscles [36], interval training is another aerobic training modality that is usually based on a maximal cardiopulmonary exercise test performed on a stationary bike for patients who are unable to perform or sustain high-intensity exercise due to dyspnea or fatigue [7]. Peripheral muscle dysfunction and its consequences have been reported in COPD patients, and resistance training aimed to train different aspects of muscle function such as strength and endurance, proving beneficial for COPD patients [37,38]. Upper limb strength and endurance can impact people’s abilities to perform daily activities, but the effect on levels of exercise capacity, dyspnea, or health-related quality of life was unclear [39].

## 3. Adjuncts That Enhance the Effects of Physical Training

Damage to the lungs of COPD patients may lead to gas exchange disturbances, which limit the capacity of patients to engage in exercise training of sufficient intensity [40]. Therefore, supplemental oxygen and the use of noninvasive ventilation should have beneficial effects. Indeed, this is the case, with previous reports showing that supplemental oxygen in COPD patients improves their exercise capacity and alleviates dyspnea [41,42]. Moreover, supplemental oxygen improved exercise tolerance, which enabled COPD patients to achieve high-intensity exercise during pulmonary rehabilitation [43]. Similarly, randomized controlled cross-over trial results concluded that supplemental oxygen generally enhanced exercise capacity as well as exercise-induced hypoxemia in COPD patients [44]. However, others indicated there are no additional benefits of training with supplemental oxygen compared with room air in terms of exercise capacity, tolerance, or health status [45,46,47], suggesting that exercise programs could be performed in venues where supplemental oxygen is not available for COPD patients, enabling pulmonary rehabilitation to be more widely accessible. These discrepant findings should be investigated in future large multicenter studies to improve the quality of data. The use of noninvasive ventilation (NIV) is beneficial in COPD patients. For instance, additional NIV enhanced quality of life and exercise endurance and relieved dyspnea in COPD patients [48]. Meanwhile, it has also been shown that NIV significantly reduces hypercapnia, time to hospital readmission, and mortality risk [49,50,51].

Apart from noninvasive ventilation, breathing exercises such as diaphragmatic breathing and pursed-lips breathing might be considered for those patients who are unable to exercise, possibly enhancing pulmonary function, exercise endurance, dyspnea, quality of life, and respiratory muscle strength of COPD patients [52]. A systematic review suggested breathing exercises are an effective tool to improve inspiratory muscle strength and 6MWT results in COPD patients [53]. A randomized controlled trial showed that the pursed-lips breathing exercise, inhaler training enhanced breathing exercise, and inhaler usage skills reduce the negative effects of COPD, alleviate the severity of dyspnea, and improve the quality of life of COPD patients [54]. Moreover, the active cycle of breathing techniques was found to effectively improve the sputum production and cough efficiency in COPD patients [55]. More research and quantitative analyses are needed to confirm the effectiveness of breath exercises on different aspects of COPD patients.

In addition, the use of pharmacological adjuncts during the course of PR might improve the effects of exercise training. For instance, a randomized study with 30 patients suggested that a combination of long-acting bronchodilators and endurance and strength training increased the 6MWD by 42m more compared with long-acting bronchodilators alone [56]. And there is suggested evidence that the administration of androgens in the context of exercise training enhances exercise capacity and muscle strength [57]. And supplement of creatine, growth hormone, and polyunsaturated fatty acids during exercise training might improve muscle function [58].

## 4. Acupuncture Techniques for Pulmonary Rehabilitation

Acupuncture therapy (AT)—derived from Traditional Chinese Medicine (TCM) and based on the same principles of Tai Chi that yin and yang is viewed in the universe as dualistic forces that must be balanced to create harmony and health [59]—is one of the most popular treatments in alternative medicine. AT uses different techniques, including the well-known insertion of needles, heat stimulation, electricity (electroacupuncture or acupoint transcutaneous electrical nerve stimulation (AcuTENS)), and digital pressure to stimulate specific areas of the body surface, or acupuncture points. AT has been traditionally performed to restore health, including respiratory diseases like COPD. Here, we summarize the effectiveness of these techniques in patients with COPD.

There is increasing evidence to show that acupuncture is an effective, adjunctive non-pharmacological treatment to enhance quality of life as well as pulmonary function in COPD patients. For instance, in randomized multicenter sham-controlled trial studies and meta-analysis reports, AT used as an adjunctive therapy alleviated dyspnea and improved the life quality of COPD patients [60,61,62,63]. AT was also found to improve the nutritional state, muscle stress, and prognosis of COPD patients [64]. Additionally, in an in vivo rodent study, both manual AT and electro-AT treatment were reported to attenuate inflammatory response to protect lung function via either increasing HDAC2 expression or modifying cholinergic anti-inflammatory pathways, respectively [65,66].

Furthermore, studies have suggested that the integration of AT with Chinese herb medicines or combined with internal–external therapy can improve therapy effects or reduce side effects in COPD patients. In a randomized controlled trial (RTC) study, Li et al. found *Bufei Yishen* granules combined with external applications of herbal paste to acupoints were a safe and effective therapy for stable COPD and showed beneficial effects compared to theophylline on the frequency of acute exacerbations, scores of symptoms, dyspnea grade, and six-minute walking distance [67]. This finding was adopted with the guidelines and is the first RCT article in the field of prevention and treatment of COPD with TCM interventions. Consistently, further study illustrated that *Bufei Yishen* and acupoint sticking therapy enhanced pulmonary function and lung pathological impairment, potentially by positively regulating the pulmonary surfactant protein expressions related to airway limitation [68,69]. Similarly, *Bufei Yishen* granules with electroacupuncture-integrated treatment had superior effects for reducing inflammatory response and improving lung function through the TLR-4/NF-κB pathway [70]. Given that the evidence is of low or medium quality, the effects of the combination of AT and herbal medicines in COPD patients remain inconclusive in comparation with AT alone. More abundant evidence proved by trained professionals with specific acupuncture skills and the pathology of COPD are needed before definitive conclusions can be drawn regarding treatment outcomes with AT versus herbal medicines or AT added to herbal medicines versus AT alone for patients with COPD.

## 5. Nutritional Support and Pulmonary Rehabilitation

Loss of body weight and fat-free mass have been commonly found in COPD patients and became a poor prognostic parameter [71]. Low body mass index (BMI) and fat-free mass are related with decreased exercise capacity, muscle weakness, and poor health status [72,73]. A meta-analysis study with at least a 2-week duration of nutritional interventions suggested that following nutritional supplementation, significant weight gain was found in the total COPD population compared to the placebo. In malnourished patients, targeted nutritional supplementation, especially calorific food, showed larger gains of weight and furthermore improved physical performance ability and quality of life [74]. This was supported with another meta-analyses which showed that the nutritional intervention resulted in a significant increase in energy intake above baseline and was accompanied by a significant increase in body weight in COPD [75].Moreover, there is suggested evidence that intakes of fruit, vegetables, dietary fibers, vitamins C and E, polyphenols, and β-carotene were individually associated with lower COPD risk, while an unhealthy diet style was related to an increased risk of COPD [76]. And oral nutritional supplements (ONS) could also increase body weight and exercise performance [77,78]. Individual nutritional therapy and collaborative exercise training might be an effective intervention, particularly handling COPD in malnourished patients.

## 6. Self-Management Education for COPD Patients

Self-management education, aiming to promote self-efficacy to change behavior, is also a core component of pulmonary rehabilitation. After training courses, patients might change behaviors, including smoking cessation, increase in physical activity, and improved adherence to medication [79]. Patient self-management education focuses on attitudes and awareness, including improving a patient’s own self-competence, helping patients identify problems, and teaching useful skills to solve disease-related problems [80]. Self-management education has proven beneficial in improving health-related quality of life (HRQoL) in patients and reduced hospital admissions [81]. A well-designed prospective trial proved that reduced admissions for exacerbations, decreased admissions for other health problems, and fewer emergency department visits were found among patients undergoing self-management education [82]. However, a randomized single-site clinical trial that combined transition and long-term self-management support showed significantly greater COPD-related hospitalizations and emergency department visits, without improvement in quality of life [83]. A survey report identified a strong interest in disease-focused education; a lack of prior instruction in specific self-management strategies for COPD and self-management interventions that target the multiple behavioral and emotional needs of COPD patients are needed [84]. Further work is necessary to evaluate possible effects of self-management in COPD.

## 7. Psychosocial Support for COPD Patients

Depression and anxiety are the major psychological characteristics in COPD patients, which compromise their ability to cope with the disease. Social care helps patients participate in working life by providing a tailored initiation of services. For older patients, it is imperative to provide possible home care or services for severe disability, the provision of therapeutic appliances/assistive technologies, and advice on social services and institutions of social care [63]. Studies suggested that psychological testing showed a reduction in depression and anxiety, together with other negative emotions including irritability, hostility, anger, fear, loneliness, hypochondriasis, hysteria, psychopathic deviance, dissatisfaction, and negative self-concept, thereby resulting in an improvement in personality styles, attitudes, and the ability to deal with the disease [85]. The meta-analysis of randomized controlled trials suggested psychosocial intervention, as an additional useful tool, has the potential to improve both psychological and physical outcomes in COPD patients [86]. Another study reported that psychosocial interventions applied in advanced COPD underline the roles of self-efficacy, telehealth, and physical activity in physical and mental health preservation [87].

## 8. Long-Term Benefits of Pulmonary Rehabilitation

Comprehensive pulmonary rehabilitation programs achieve improvements in symptoms, quality of life, and exercise capacity and can reduce hospitalization, but the benefits decrease over time. In order to observe the long-term effects of PR, the follow-up PR programs were undertaken by pertinent studies. A 2-year follow up of patient-reported outcome measures after the completion of an 8-week pulmonary rehabilitation program suggested improvements of dyspnea, depression. Stress symptoms at 8 weeks were not maintained at 2 years but provided sustained improvement in anxiety and quality of life [88]. Another 24-month PR program showed progressively increased quality of life, dyspnea, and exercise tolerance, and it reduced cardiovascular risk factors in COPD patients [89]. Visits over 5 years for COPD patients who completed PR found the dyspnea and depression scores increased progressively over the 5 years [90]. In a feasible community-based maintenance exercise program, they reported clinically preserved improvements of exercise capacity and health-related quality of life at 6 months and 1 year after PR compared to the baseline in COPD patients [91].

## 9. Conclusions

There is currently no treatment that is specifically targeted for the various physical, emotional, and social symptoms that COPD patients experience. As a result, it is essential to carry out an extensive, customized intervention provided as a customized pulmonary rehabilitation (PR) program. Clinical advancement and high PR effectiveness among COPD patients are presented in this review. According to the findings, the majority of patients could be expected to experience improvements after completing the PR program in terms of various acupuncture treatments and exercise training models. Exercise tolerance or other functional parameters were improved with additional oxygen support and noninvasive ventilation. Self-management education changes attitudes and awareness to bring about an increase in self-efficiency. Psychosocial care would be helpful to improve concurrent morbidities such as depression and anxiety. The PR program will increase the patient’s level of personal autonomy and enable them to function in society to the fullest extent possible. Future research on clinical improvement and effectiveness utilizing additional PR outcomes is still necessary.

## Data Availability

Not applicable.

## References

[1] Rao W., Wang S., Duleba M., Niroula S., Goller K., Xie J., Mahalingam R., Neupane R., Liew A.-A., Vincent M. (2020). Regenerative Metaplastic Clones in COPD Lung Drive Inflammation and Fibrosis. Cell.

[2] Wang C., Zhou J., Wang J., Li S., Fukunaga A., Yodoi J., Tian H. (2020). Progress in the mechanism and targeted drug therapy for COPD. Signal Transduct. Target. Ther..

[3] Guo P., Li R., Piao T.H., Wang C.L., Wu X.L., Cai H.Y. (2022). Pathological Mechanism and Targeted Drugs of COPD. Int. J. Chronic Obstr. Pulm. Dis..

[4] Linden D., Guo-Parke H., Coyle P.V., Fairley D., McAuley D.F., Taggart C.C., Kidney J. (2019). Respiratory viral infection: A potential “missing link” in the pathogenesis of COPD. Eur. Respir. Rev..

[5] Tanabe N., Sato S., Muro S., Shima H., Oguma T., Tanimura K., Sato A., Hirai T. (2019). Regional lung deflation with increased airway volume underlies the functional response to bronchodilators in chronic obstructive pulmonary disease. Physiol. Rep..

[6] Cordova-Rivera L., Gibson P.G., Gardiner P.A., McDonald V.M. (2018). Physical activity associates with disease characteristics of severe asthma, bronchiectasis and COPD. Respirology.

[7] Spruit M.A., Singh S.J., Garvey C., ZuWallack R., Nici L., Rochester C., Hill K., Holland A.E., Lareau S.C., Man W.D.-C. (2013). An official American Thoracic Society/European Respiratory Society statement: Key concepts and advances in pulmonary rehabilitation. Am. J. Respir. Crit. Care Med..

[8] Waschki B., Spruit M.A., Watz H., Albert P.S., Shrikrishna D., Groenen M., Smith C., Man W.D.-C., Tal-Singer R., Edwards L.D. (2012). Physical activity monitoring in COPD: Compliance and associations with clinical characteristics in a multicenter study. Respir. Med..

[9] Carrai R., Scano G., Gigliotti F., Romagnoli I., Lanini B., Coli C., Grippo A. (2012). Prevalence of limb muscle dysfunction in patients with chronic obstructive pulmonary disease admitted to a pulmonary rehabilitation centre. Clin. Neurophysiol..

[10] Nici L., Donner C., Wouters E., Zuwallack R., Ambrosino N., Bourbeau J., Carone M., Celli B., Engelen M., Fahy B. (2006). American Thoracic Society/European Respiratory Society Statement on Pulmonary Rehabilitation. Am. J. Respir. Crit. Care Med..

[11] Gendron L.M., Nyberg A., Saey D., Maltais F., Lacasse Y. (2018). Active mind-body movement therapies as an adjunct to or in comparison with pulmonary rehabilitation for people with chronic obstructive pulmonary disease. Cochrane Database Syst. Rev..

[12] Wayne P.M., Kaptchuk T.J. (2008). Challenges Inherent to*T’ai Chi* Research: Part I—*T’ai Chi* as a Complex Multicomponent Intervention. J. Altern. Complement. Med..

[13] Leung R.W., McKeough Z.J., Alison J.A. (2013). Tai Chi as a form of exercise training in people with chronic obstructive pulmonary disease. Expert Rev. Respir. Med..

[14] Ngai S.P., Jones A.Y., Tam W.W.S. (2016). Tai Chi for chronic obstructive pulmonary disease (COPD). Cochrane Database Syst. Rev..

[15] Chan A.W.K., Lee A., Suen L.K.P., Tam W.W.S. (2010). Effectiveness of a Tai chi Qigong program in promoting health-related quality of life and perceived social support in chronic obstructive pulmonary disease clients. Qual. Life Res..

[16] Chan A.W., Lee A., Suen L.K., Tam W.W. (2011). Tai chi Qigong improves lung functions and activity tolerance in COPD clients: A single blind, randomized controlled trial. Complement. Ther. Med..

[17] Leung R.W.M., McKeough Z.J., Peters M.J., Alison J.A. (2012). Short-form Sun-style t’ai chi as an exercise training modality in people with COPD. Eur. Respir. J..

[18] Yan J.-H., Guo Y.-Z., Yao H.-M., Pan L. (2013). Effects of Tai Chi in Patients with Chronic Obstructive Pulmonary Disease: Preliminary Evidence. PLoS ONE.

[19] Niu R., He R., Luo B.-L., Hu C. (2014). The Effect of Tai Chi on Chronic Obstructive Pulmonary Disease: A Pilot Randomised Study of Lung Function, Exercise Capacity and Diaphragm Strength. Heart Lung Circ..

[20] Guo C., Xiang G., Xie L., Liu Z., Zhang X., Wu Q., Li S., Wu Y. (2020). Effects of Tai Chi training on the physical and mental health status in patients with chronic obstructive pulmonary disease: A systematic review and meta-analysis. J. Thorac. Dis..

[21] Zhang H., Li J.-S., Yu X.-Q., Li S.-Y., Upur H., Xie Y., Wang Y.-F., Li F.-S., Wang M.-H. (2017). An evaluation of activity tolerance, patient-reported outcomes and satisfaction with the effectiveness of pulmonary daoyin on patients with chronic obstructive pulmonary disease. Int. J. Chronic Obstr. Pulm. Dis..

[22] Yu X.-Q., Li J.-S., Li S.-Y., Xie Y., Wang M.-H., Zhang H.-L., Wang H.-F., Wang Z.-W. (2013). Functional and psychosocial effects of pulmonary Daoyin on patients with COPD in China: Study protocol of a multicenter randomized controlled trial. J. Integr. Med..

[23] Brown R.P., Gerbarg P.L. (2009). Yoga breathing, meditation, and longevity. Ann. N. Y. Acad. Sci..

[24] Kasse C., Prado E., Raso V., Scharlach R. (2014). Hatha yoga on body balance. Int. J. Yoga.

[25] Harinath K., Malhotra A.S., Pal K., Prasad R., Kumar R., Kain T.C., Rai L., Sawhney R.C., Hendriks T., de Jong J. (2004). Effects of Hatha yoga and Omkar meditation on cardiorespiratory performance, psychologic profile, and melatonin secretion. J. Altern. Complement. Med..

[26] Fulambarker A., Farooki B., Kheir F., Copur A.S., Srinivasan L., Schultz S. (2012). Effect of yoga in chronic obstructive pulmonary disease. Am. J. Ther..

[27] Pomidori L., Campigotto F., Amatya T.M., Bernardi L., Cogo A. (2009). Efficacy and tolerability of yoga breathing in patients with chronic obstructive pulmonary disease: A pilot study. J. Cardiopulm. Rehabil. Prev..

[28] Ranjita R., Hankey A., Nagendra H., Mohanty S. (2016). Yoga-based pulmonary rehabilitation for the management of dyspnea in coal miners with chronic obstructive pulmonary disease: A randomized controlled trial. J. Ayurveda Integr. Med..

[29] Troosters T., Casaburi R., Gosselink R., Decramer M. (2005). Pulmonary rehabilitation in chronic obstructive pulmonary disease. Am. J. Respir. Crit. Care Med..

[30] Soni R., Munish K., Singh K., Singh S. (2012). Study of the effect of yoga training on diffusion capacity in chronic obstructive pulmonary disease patients: A controlled trial. Int. J. Yoga.

[31] Donesky-Cuenco D., Nguyen H.Q., Paul S.M., Carrieri-Kohlman V. (2009). Yoga therapy decreases dyspnea-related distress and improves functional performance in people with chronic obstructive pulmonary disease: A pilot study. J. Altern. Complement. Med..

[32] Puhan M.A., Gimeno-Santos E., Cates C.J., Troosters T. (2016). Pulmonary rehabilitation following exacerbations of chronic obstructive pulmonary disease. Cochrane Database Syst. Rev..

[33] Breyer M.-K., Breyer-Kohansal R., Funk G.-C., Dornhofer N., Spruit M.A., Wouters E.F., Burghuber O.C., Hartl S. (2010). Nordic Walking improves daily physical activities in COPD: A randomised controlled trial. Respir. Res..

[34] Leung R.W., Alison J.A., McKeough Z., Peters M. (2010). Ground walk training improves functional exercise capacity more than cycle training in people with chronic obstructive pulmonary disease (COPD): A randomised trial. J. Physiother..

[35] McNamara R.J., McKeough Z.J., McKenzie D.K., Alison J.A. (2012). Water-based exercise in COPD with physical comorbidities: A randomised controlled trial. Eur. Respir. J..

[36] Man W.D., Soliman M.G., Gearing J., Radford S.G., Rafferty G.F., Gray B.J., Polkey M.I., Moxham J. (2003). Symptoms and quadriceps fatigability after walking and cycling in chronic obstructive pulmonary disease. Am. J. Respir. Crit. Care Med..

[37] American College of Sports Medicine Position Stand (2009). Progression models in resistance training for healthy adults. Med. Sci. Sports Exerc..

[38] O’Shea S.D., Taylor N.F., Paratz J.D. (2009). Progressive resistance exercise improves muscle strength and may improve elements of performance of daily activities for people with COPD: A systematic review. Chest.

[39] Janaudis-Ferreira T., Hill K., Goldstein R.S., Robles-Ribeiro P., Beauchamp M.K., Dolmage T.E., Wadell K., Brooks D. (2011). Resistance arm training in patients with COPD: A Randomized Controlled Trial. Chest.

[40] O’Donnell D.E., Revill S.M., Webb K.A. (2001). Dynamic Hyperinflation and Exercise Intolerance in Chronic Obstructive Pulmonary Disease. Am. J. Respir. Crit. Care Med..

[41] Wadell K.H.-L.K. (2001). Physical training with and without oxygen in patients with chronic obstructive pulmonary disease and exercise-induced hypoxaemia. J. Rehabil. Med..

[42] Neunhäuserer D., Reich B., Mayr B., Kaiser B., Lamprecht B., Niederseer D., Ermolao A., Studnicka M., Niebauer J. (2020). Impact of exercise training and supplemental oxygen on submaximal exercise performance in patients with COPD. Scand. J. Med. Sci. Sports.

[43] Emtner M., Porszasz J., Burns M., Somfay A., Casaburi R. (2003). Benefits of supplemental oxygen in exercise training in nonhypoxemic chronic obstructive pulmonary disease patients. Am. J. Respir. Crit. Care Med..

[44] Jarosch I., Gloeckl R., Damm E., Schwedhelm A.-L., Buhrow D., Jerrentrup A., Spruit M.A., Kenn K. (2017). Short-term Effects of Supplemental Oxygen on 6-Min Walk Test Outcomes in Patients with COPD. Chest.

[45] Garrod R., Paul E.A., Wedzicha J.A. (2000). Supplemental oxygen during pulmonary rehabilitation in patients with COPD with exercise hypoxaemia. Thorax.

[46] Alison J.A., McKeough Z.J., Leung R.W., Holland A.E., Hill K., Morris N.R., Jenkins S., Spencer L.M., Hill C.J., Lee A.L. (2019). Oxygen compared to air during exercise training in COPD with exercise-induced desaturation. Eur. Respir. J..

[47] Héraud N., Préfaut C., Durand F., Varray A. (2008). Does correction of exercise-induced desaturation by O2 always improve exercise tolerance in COPD? A preliminary study. Respir. Med..

[48] Duiverman M.L., Wempe J.B., Bladder G., Vonk J.M., Zijlstra J.G., Kerstjens H.A., Wijkstra P.J. (2011). Two-year home-based nocturnal noninvasive ventilation added to rehabilitation in chronic obstructive pulmonary disease patients: A randomized controlled trial. Respir. Res..

[49] Murphy P.B., Rehal S., Arbane G., Bourke S., Calverley P.M.A., Crook A.M., Dowson L., Duffy N., Gibson G.J., Hughes P.D. (2017). Effect of Home Noninvasive Ventilation with Oxygen Therapy vs Oxygen Therapy Alone on Hospital Readmission or Death After an Acute COPD Exacerbation: A Randomized Clinical Trial. JAMA.

[50] Köhnlein T., Windisch W., Köhler D., Drabik A., Geiseler J., Hartl S., Karg O., Laier-Groeneveld G., Nava S., Schönhofer B. (2014). Non-invasive positive pressure ventilation for the treatment of severe stable chronic obstructive pulmonary disease: A prospective, multicentre, randomised, controlled clinical trial. Lancet Respir. Med..

[51] Ambrosino N., Xie L., Ambrosino N., Xie L. (2017). The Use of Non-invasive Ventilation during Exercise Training in COPD Patients. COPD J. Chronic Obstr. Pulm. Dis..

[52] Li Y., Ji Z., Wang Y., Li X., Xie Y. (2022). Breathing Exercises in the Treatment of COPD: An Overview of Systematic Reviews. Int. J. Chronic Obstr. Pulm. Dis..

[53] Yun R., Bai Y., Lu Y., Wu X., Lee S.D. (2021). How Breathing Exercises Influence on Respiratory Muscles and Quality of Life among Patients with COPD? A Systematic Review and Meta-Analysis. Can. Respir. J..

[54] Ceyhan Y., Tekinsoy Kartin P. (2022). The effects of breathing exercises and inhaler training in patients with COPD on the severity of dyspnea and life quality: A randomized controlled trial. Trials.

[55] Shen M., Li Y., Ding X., Xu L., Li F., Lin H. (2020). Effect of active cycle of breathing techniques in patients with chronic obstructive pulmonary disease: A systematic review of intervention. Eur. J. Phys. Rehabil. Med..

[56] Weiner P., Magadle R., Berar-Yanay N., Davidovich A., Weiner M. (2000). The cumulative effect of long-acting bronchodilators, exercise, and inspiratory muscle training on the perception of dyspnea in patients with advanced COPD. Chest.

[57] Casaburi R., Bhasin S., Cosentino L., Porszasz J., Somfay A., Lewis M.I., Fournier M., Storer T.W. (2004). Effects of testosterone and resistance training in men with chronic obstructive pulmonary disease. Am. J. Respir. Crit. Care Med..

[58] Varadi R.G., Goldstein R. (2009). Pulmonary rehabilitation and pharmacotherapy. Semin. Respir. Crit. Care Med..

[59] Dundee J.W., Chestnutt W.N., Ghaly R.G., Lynas A.G. (1986). Traditional Chinese acupuncture: A potentially useful antiemetic?. BMJ.

[60] Feng J., Wang X., Li X., Zhao D., Xu J. (2016). Acupuncture for chronic obstructive pulmonary disease (COPD). Medicine.

[61] Hsieh P.-C., Yang M.-C., Wu Y.-K., Chen H.-Y., Tzeng I.-S., Hsu P.-S., Lee C.-T., Chen C.-L., Lan C.-C. (2019). Acupuncture therapy improves health-related quality of life in patients with chronic obstructive pulmonary disease: A systematic review and meta-analysis. Complement. Ther. Clin. Pract..

[62] Fernández-Jané C., Vilaró J., Fei Y., Wang C., Liu J., Huang N., Xia R., Tian X., Hu R.-X., Yu M. (2019). Filiform needle acupuncture for copd: A systematic review and meta-analysis. Complement. Ther. Med..

[63] Wang J., Li J., Yu X., Xie Y. (2018). Acupuncture Therapy for Functional Effects and Quality of Life in COPD Patients: A Systematic Review and Meta-Analysis. BioMed Res. Int..

[64] Suzuki M., Muro S., Fukui M., Ishizaki N., Sato S., Shiota T., Endo K., Suzuki T., Mitsuma T., Mishima M. (2018). Effects of acupuncture on nutritional state of patients with stable chronic obstructive pulmonary disease (COPD): Re-analysis of COPD acupuncture trial, a randomized controlled trial. BMC Complement. Altern. Med..

[65] Li J., Wu S., Tang H., Huang W., Wang L., Zhou H., Zhou M., Wang H., Li J. (2016). Long-term effects of acupuncture treatment on airway smooth muscle in a rat model of smoke-induced chronic obstructive pulmonary disease. Acupunct. Med..

[66] Zhang X.-F., Xiang S.-Y., Geng W.-Y., Cong W.-J., Lu J., Jiang C.-W., Wang K., Liu Z.-B. (2018). Electro-acupuncture regulates the cholinergic anti-inflammatory pathway in a rat model of chronic obstructive pulmonary disease. J. Integr. Med..

[67] Li J.-S., Li S.-Y., Yu X.-Q., Xie Y., Wang M.-H., Li Z.-G., Zhang N.-Z., Shao S.-J., Zhang Y.-J., Zhu L. (2011). Bu-Fei Yi-Shen granule combined with acupoint sticking therapy in patients with stable chronic obstructive pulmonary disease: A randomized, double-blind, double-dummy, active-controlled, 4-center study. J. Ethnopharmacol..

[68] Tian Y., Li Y., Li J., Xie Y., Wang M., Dong Y., Li L., Mao J., Wang L., Luo S. (2015). Bufei Yishen granule combined with acupoint sticking improves pulmonary function and morphormetry in chronic obstructive pulmonary disease rats. J. BMC Complement. Altern. Med..

[69] Tian Y., Li J., Li Y., Dong Y., Yao F., Mao J., Li L., Wang L., Luo S., Wang M. (2016). Effects of Bufei Yishen Granules Combined with Acupoint Sticking Therapy on Pulmonary Surfactant Proteins in Chronic Obstructive Pulmonary Disease Rats. BioMed Res. Int..

[70] Ma J., Tian Y., Li J., Zhang L., Wu M., Zhu L., Liu S. (2019). Effect of Bufei Yishen Granules Combined with Electroacupuncture in Rats with Chronic Obstructive Pulmonary Disease via the Regulation of TLR-4/NF-κB Signaling. Evid.-Based Complement. Altern. Med..

[71] Ferreira I.M., Brooks D., White J., Goldstein R. (2012). Nutritional supplementation for stable chronic obstructive pulmonary disease. Cochrane Database Syst. Rev..

[72] Franssen F.M.E., Broekhuizen R., Janssen P.P., Wouters E.F.M., Schols A.M.W.J. (2005). Limb muscle dysfunction in COPD: Effects of muscle wasting and exercise training. Med. Sci. Sports Exerc..

[73] Baarends E., Schols A., Mostert R., Wouters E. (1997). Peak exercise response in relation to tissue depletion in patients with chronic obstructive pulmonary disease. Eur. Respir. J..

[74] Ferreira I.M., Brooks D., Lacasse Y., Goldstein R.S., White J. (2002). Nutritional supplementation for stable chronic obstructive pulmonary disease. Cochrane Database Syst. Rev..

[75] Collins P.F., Stratton R.J., Elia M. (2012). Nutritional support in chronic obstructive pulmonary disease: A systematic review and meta-analysis. Am. J. Clin. Nutr..

[76] van Iersel L.E.J., Beijers R., Gosker H.R., Schols A. (2022). Nutrition as a modifiable factor in the onset and progression of pulmonary function impairment in COPD: A systematic review. Nutr. Rev..

[77] Steiner M.C., Barton R.L., Singh S.J., Morgan M.D. (2003). Nutritional enhancement of exercise performance in chronic obstructive pulmonary disease: A randomised controlled trial. Thorax.

[78] Collins P.F., Yang I.A., Chang Y.C., Vaughan A. (2019). Nutritional support in chronic obstructive pulmonary disease (COPD): An evidence update. J. Thorac. Dis..

[79] Gloeckl R., Schneeberger T., Jarosch I., Kenn K. (2018). Pulmonary Rehabilitation and Exercise Training in Chronic Obstructive Pulmonary Disease. Dtsch. Arztebl. Int..

[80] Hessler D.M., Fisher L., Bowyer V., Dickinson L.M., Jortberg B.T., Kwan B., Fernald D.H., Simpson M., Dickinson W.P. (2019). Self-management support for chronic disease in primary care: Frequency of patient self-management problems and patient reported priorities, and alignment with ultimate behavior goal selection. BMC Fam. Pract..

[81] Lenferink A., Brusse-Keizer M., van der Valk P.D., Frith P.A., Zwerink M., Monninkhof E.M., van der Palen J., Effing T.W. (2017). Self-management interventions including action plans for exacerbations versus usual care in patients with chronic obstructive pulmonary disease. Cochrane Database Syst. Rev..

[82] Bourbeau J., Julien M., Maltais F., Rouleau M., Beaupré A., Bégin R., Renzi P., Nault D., Borycki E., Schwartzman K. (2003). Reduction of hospital utilization in patients with chronic obstructive pulmonary disease: A disease-specific self-management intervention. Arch. Intern. Med..

[83] Aboumatar H., Naqibuddin M., Chung S., Chaudhry H., Kim S.W., Saunders J., Bone L., Gurses A.P., Knowlton A., Pronovost P. (2019). Effect of a Hospital-Initiated Program Combining Transitional Care and Long-term Self-management Support on Outcomes of Patients Hospitalized with Chronic Obstructive Pulmonary Disease: A Randomized Clinical Trial. JAMA.

[84] Mathew A.R., Guzman M., Bridges C., Yount S., Kalhan R., Hitsman B. (2019). Assessment of Self-Management Treatment Needs among COPD Helpline Callers. COPD.

[85] Güell R., Resqueti V., Sangenis M., Morante F., Martorell B., Casan P., Guyatt G.H. (2006). Impact of pulmonary rehabilitation on psychosocial morbidity in patients with severe COPD. Chest.

[86] Farver-Vestergaard I., Danielsen J.T.T., Løkke A., Zachariae R. (2022). Psychosocial Intervention in Chronic Obstructive Pulmonary Disease: Meta-Analysis of Randomized Controlled Trials. Psychosom. Med..

[87] Rzadkiewicz M., Nasiłowski J. (2019). Psychosocial Interventions for Patients with Severe COPD-An Up-to-Date Literature Review. Medicina.

[88] Yohannes A.M., Dryden S., Casaburi R., Hanania N.A. (2021). Long-Term Benefits of Pulmonary Rehabilitation in Patients with COPD: A 2-Year Follow-Up Study. Chest.

[89] Reis L.F., Guimaraes F.S., Fernandes S.J., Cantanhede L.A., Dias C.M., Lopes A.J., De Menezes S.L. (2013). A long-term pulmonary rehabilitation program progressively improves exercise tolerance, quality of life and cardiovascular risk factors in patients with COPD. Eur. J. Phys. Rehabil. Med..

[90] Candemir İ., Ergün P., Şahin M.E. (2021). Maintenance of pulmonary rehabilitation benefits in patients with COPD: Is a structured 5-year follow-up program helpful?. Turk. J. Med. Sci..

[91] Beauchamp M.K., Francella S., Romano J.M., Goldstein R.S., Brooks D. (2013). A novel approach to long-term respiratory care: Results of a community-based post-rehabilitation maintenance program in COPD. Respir. Med..

